# Interferon-α curbs production of interleukin-22 by human peripheral blood mononuclear cells exposed to live *Borrelia burgdorferi*

**DOI:** 10.1111/jcmm.12634

**Published:** 2015-07-08

**Authors:** Anika Berner, Malte Bachmann, Josef Pfeilschifter, Peter Kraiczy, Heiko Mühl

**Affiliations:** a Pharmazentrum Frankfurt/ZAFES, University Hospital Goethe-University FrankfurtFrankfurt am Main, Germany; b Institute of Medical Microbiology and Infection Control, University Hospital Goethe-University FrankfurtFrankfurt am Main, Germany

**Keywords:** interleukin-1, interleukin-22, interferon-α, inflammation, host defence, immune response, *Borrelia burgdorferi*, peripheral blood mononuclear cells

## Abstract

Cytokine networks initiated by means of innate immunity are regarded as a major determinant of host defence in response to acute infection by bacteria including *Borrelia burgdorferi*. Herein, we demonstrate that interferon (IFN)-α, either endogenously produced after exposure of cells to toll-like receptor-9-activating CpG oligonucleotides or provided as recombinant cytokine, weakens activation of the anti-bacterial interleukin (IL)-1/IL-22 axis in human peripheral blood mononuclear cells exposed to viable *B. burgdorferi*. As IFN-α has been related to pathological dissemination of the spirochaete, data suggest an immunoregulatory role of type I IFN in this context that is able to significantly modify cytokine profiles thereby possibly determining early course of *B. burgdorferi* infection.

## Introduction

*Borrelia burgdorferi* is a spirochaete of the *B. burgdorferi sensu lato complex* causing Lyme borreliosis, a bacterial infectious disease transmitted by tick bites that, if insufficiently treated, may progress to chronic inflammatory manifestations such as Acrodermatitis chronica atrophicans, neuroborreliosis or Lyme arthritis. Notably, despite adequate antibiotic pharmacotherapy, a small fraction of hard-to-treat patients will proceed to chronicity and thus may develop aforementioned serious disease entities 1–3.

Course of infection and dissemination of *B. burgdorferi* is supposed to be affected by initial activation of innate immunity and subsequent cytokine cascades mediating anti-bacterial inflammatory responses. Cellular sensors known to be activated by *B. burgdorferi* include toll-like receptor (TLR)-2, TLR3, TLR7-9, and nucleotide organization domain-2 3. As detected by analysis of exposed human peripheral blood mononuclear cells (PBMC) in culture, *B. burgdorferi* is a broad inducer of inflammatory cytokines. Among the cytokines up-regulated by *B. burgdorferi*, interleukin (IL)-1β has an outstanding capacity to initiate inflammatory responses supposedly strengthening host defence 4–7. In fact, up-regulated tissue IL-1β has been detected in *Erythema migrans* lesions of patients displaying acute flu-like symptoms after infection by *B. burgdorferi* 8. Secretion of biological active IL-1β depends largely on caspase-1 9, a protease activated under the influence of the spirochaete 10. Recently, we reported on expression of IL-22 by human PBMC exposed to live *B. burgdorferi* 6. Production of this key anti-bacterial and tissue-protective cytokine 11 is largely dependent on IL-1β biological activity 6, an observation emphasizing the pivotal role of the latter cytokine in that context.

Type I interferons (IFN) are up-regulated under the influence of *B. burgdorferi* 5,7. As type I IFN display a substantial immunoregulatory potential 12,13, we set out to investigate herein, effects of IFN-α on IL-22 production by PBMC under the influence of live *B. burgdorferi*.

## Materials and methods

### Bacterial isolates and culture conditions

Culture conditions were described previously 6. Briefly, cerebrospinal-derived *B. burgdorferi* 297 was grown at 33°C for 4 days up to cell densities of 1 × 10^7^/ml in modified Barbour–Stoenner–Kelly medium. Thereafter, spirochaetes were used for cocultivation with PBMC.

### Isolation and stimulation of human PBMC

Healthy donors had abstained from taking drugs for 2 weeks prior to the study. In this study PBMC from nine different donors were investigated. PBMC were freshly isolated from peripheral blood using Histopaque-1077 (Sigma-Aldrich, Taufkirchen, Germany) according to the manufacturer’s instructions. For cultivation, PBMC were routinely resuspended in Roswell Park Memorial Institute (RPMI) 1640 supplemented with 10 mM 4-(2-hydroxyethyl)-1-piperazineethanesulfonic acid (HEPES) and 1% human serum (Invitrogen by Life Technologies, Darmstadt, Germany) and seeded at 3 × 10^6^ cells/ml in round-bottom polypropylene tubes (Greiner, Frickenhausen, Germany). The protocol was approved by the ‘Ethik Kommission’ of the University Hospital Goethe-University Frankfurt. PBMC were activated by *B. burgdorferi* 297 (at 0.1 MOI), CpG type A (ODN2216: 5′-ggGGGACGATCGTCgggggg-3′; InvivoGen, San Diego, CA, USA), recombinant human IFN-α (R&D Systems, Abingdon, UK), recombinant B18R (eBioscience, Frankfurt, Germany) or anti-human CD3 antibody (Biozol, Eching, Germany).

### Detection of IL-22, IL-1β, and IFN-α mRNA by real-time PCR

Total RNA was isolated using TRI-Reagent (Sigma-Aldrich, Taufenkirchen, Germany) and transcribed using random hexameric primers and Moloney virus reverse transcriptase (Applied Biosystems, Weiterstadt, Germany). Pre-developed assay reagents were obtained from Applied Biosystems: IL-22 (FAM; #Hs00220924_m1), glyceraldehyde-phosphate dehydrogenase (GAPDH; VIC; #4310884E). Assay-mix was used from Thermoscientific (Langenselbold, Germany). Real-time PCR was performed on AbiPrism 7500 Fast Sequence Detector (Applied Biosystems, Darmstadt, Germany): Detection of the dequenched probe, calculation of threshold cycles (Ct values) and data analysis were performed by the Sequence Detector software. Relative changes in mRNA expression compared to unstimulated control and normalized to GAPDH were quantified by the 2^−ddCt^ method.

### Analysis of cytokine release by ELISA

Interleukin-8 (Pharmingen/BD Biosciences, Heidelberg, Germany), IL-1β, IL-22 (R&D Systems) and IFN-α (eBioscience) in cell-free culture supernatants were determined by ELISA according to the manufacturers’ instructions.

### Statistical analysis

Data are shown as means ± SEM and are presented as pg/ml, ng/ml, as (% of *B. burgdorferi*) or as fold-induction. Using raw data, statistical analysis was performed by one-way ANOVA with post hoc Bonferroni correction (GraphPad 5.0; GraphPad Inc., La Jolla, CA, USA).

## Results

**Figure 1:**
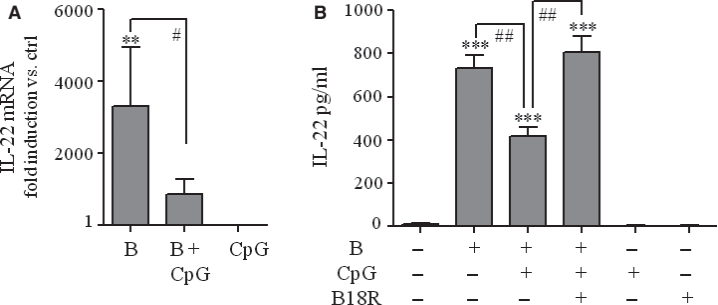
(A) PBMC were kept as an unstimulated control or were exposed to *Borrelia burgdorferi* (MOI = 0.1) in the presence or absence of CpG-ODN (1 μg/ml) or with CpG-ODN (1 μg/ml) alone. After 24 hrs, total RNA was isolated and IL-22 mRNA expression was determined by real-time PCR. IL-22 mRNA was normalized to that of GAPDH and is shown as mean fold-induction compared to unstimulated control ± SEM (*n* = 3); **P < 0.01 compared with unstimulated control, ^#^*P* < 0.05. (B) PBMC were kept as an unstimulated control or were exposed to *B. burgdorferi* (MOI = 0.1) in presence or absence of CpG-ODN (1 μg/ml) or with CpG-ODN (1 μg/ml) alone. Where indicated, B18R (0.1 μg/ml) was added. After 65 hrs, release of IL-22 was determined by ELISA. Data are expressed as means ± SEM (*n* = 4); ***P < 0.001 compared with unstimulated control, ^##^*P* < 0.01. B, denotes exposure of PBMC to *B. burgdorferi*.

Characterizing the production of IL-22 by *B. burgdorferi*-stimulated PBMC, we recently reported on inhibition of secretion of this cytokine by exposure of cells to CpG oligonucleotides (CpG-ODN) 6. Herein, we set out to further investigate this regulatory path. Data shown in Figure 1A confirm modulatory properties of CpG-ODN in this context and extend our previous observations onto the level of IL-22 mRNA expression. Regulation of PBMC activation by the CpG-ODN used herein is likely *via* effects on plasmacytoid dendritic cells that efficiently express TLR9. This receptor connects to robust production of type I IFN by PBMC, foremost IFN-α 14,15. To further associate CpG-ODN effects with production of IFN-α, PBMC were activated by *B. burgdorferi* cells in the presence of CpG-ODN and recombinant B18R. This latter molecule is a *Vaccinia* virus-encoded soluble type I IFN receptor with antagonistic activity on type I IFN biological activity 16. In fact, coincubation with B18R was able to completely overcome inhibitory effects of CpG-ODN on *B. burgdorferi*-induced IL-22, pointing at a role for type I IFN in this context (Fig. 1B). As expected, high levels of IFN-α were detectable in PBMC culture supernatants under the influence of CpG-ODN at 1 μg/ml [stimulation with CpG-ODN alone (65 hrs, 10.57 ± 3.47 ng/ml of IFN-α, *n* = 3, *P* < 0.01 *versus* unstimulated control); stimulation with *B. burgdorferi* plus CpG-ODN (65 hrs, 10.59 ± 3.57 ng/ml of IFN-α, *n* = 3, *P* < 0.01 *versus* unstimulated control)]. Notably, IFN-α protein secretion was barely detectable in unstimulated PBMC or those exposed to *B. burgdorferi* at 0.1 MOI as single stimulus (<15 pg/ml). This latter observation should relate to the low spirochaete concentration that was specifically selected herein for PBMC stimulation.

**Figure 2:**
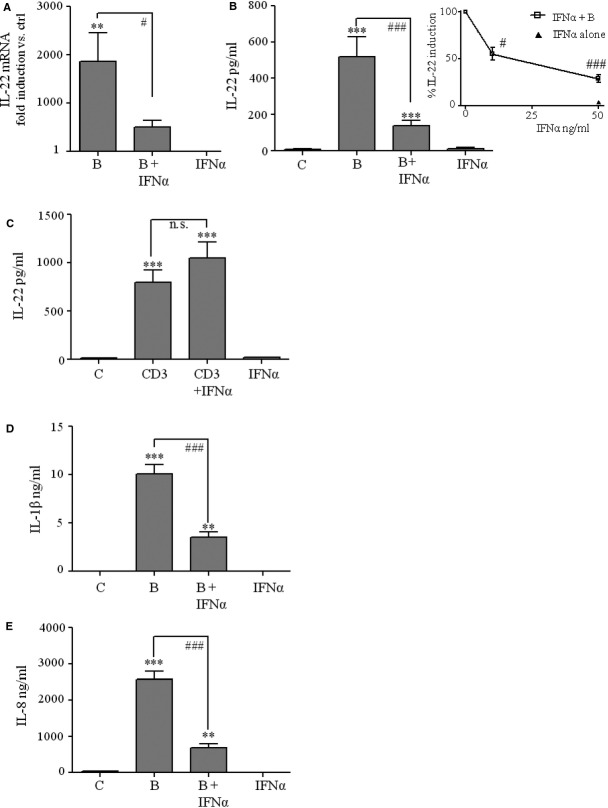
(A) PBMC were kept as an unstimulated control or exposed to *Borrelia burgdorferi* (MOI = 0.1) in presence or absence of IFN-α (5 ng/ml) or with IFN-α (5 ng/ml) alone. After 24 hrs, total RNA was isolated and IL-22 mRNA expression was determined by realtime PCR. IL-22 mRNA was normalized to that of GAPDH and is shown as mean fold-induction compared to unstimulated control ± SEM (*n* = 4); **P < 0.01 compared with unstimulated control, ^#^*P* < 0.05. (B) PBMC were kept as unstimulated control or exposed to *B. burgdorferi* (MOI = 0.1) in presence or absence of IFN-α (50 ng/ml) or with IFN-α (50 ng/ml) alone. After 65 hrs, release of IL-22 was determined by ELISA. Data are expressed as means ± SEM (*n* = 8); ***P < 0.001 compared with unstimulated control, ^###^*P* < 0.001. Inset: PBMC were kept as unstimulated control, were exposed to *B. burgdorferi* (MOI = 0.1) in the presence or absence of the indicated concentrations of IFN-α (10 or 50 ng/ml) or with IFN-α (50 ng/ml) alone. After 65 hrs, release of IL-22 was determined by ELISA. Data (% of *B. burgdorferi* alone) are expressed as means ± SEM (*n* = 8); ^##^*P* < 0.05, ^###^*P* < 0.001 compared with *B. burgdorferi* alone. (C) PBMC were kept as an unstimulated control or were activated by anti-CD3 (5 μg/ml) in the presence or absence of IFN-α (50 ng/ml) or by IFN-α (50 ng/ml) alone. After 65 hrs, release of IL-22 was determined by ELISA. Data are expressed as means ± SEM (*n* = 4); ***P < 0.001 compared with unstimulated control. n.s. denotes, not significant. (D and E) PBMC were kept as an unstimulated control or exposed to *B. burgdorferi* (MOI = 0.1) in presence or absence of IFN-α (50 ng/ml) or with IFN-α (50 ng/ml) alone. After 65 hrs, release of IL-1β (D) or IL-8 (E) was determined by ELISA. Data are expressed as means ± SEM (*n* = 8); **P < 0.01; ***P < 0.001 compared with unstimulated control; ^###^*P* < 0.001. C or B, denote cultivation of unstimulated PBMC or exposure of PBMC to *B. burgdorferi*, respectively.

To further evaluate a potential regulatory function of IFN-α on *B. burgdorferi*-stimulated IL-22, PBMC activation was analysed under the influence of this type I IFN. In fact, Figure 2A demonstrates significant suppression of *B. burgdorferi*-induced IL-22 mRNA expression by IFN-α. Modulation of IL-22 mRNA likewise translated to the level of cytokine secretion (Fig. 2B). Dose-response analysis revealed that effects of IFN-α were optimal at 50 ng/ml (Fig. 2B, inset), which was used in further experiments. Modulation of IL-22 secretion by IFN-α essentially connected to the induction of innate immunity, as this regulatory path was not detectable in case of IL-22 production in response to polyclonal T cell activation using an agonistic anti-CD3 antibody (Fig. 2C). Notably, type I IFN, namely IFN-β, has previously been reported to inhibit spontaneous release of IL-22 from otherwise unstimulated CD4^+^ T cells obtained from multiple sclerosis patients 17. In this context it is noteworthy that memory helper CD4^+^ and cytotoxic CD8^+^ T cells but also naïve CD4^+^ T cells exposed to an inflammatory environment can produce ample amounts of cytokines in an antigen-/T cell receptor-independent manner 18,19. In accord with this notion, we recently identified T cells, mainly CD4^+^ but also CD8^+^ T cells, as chief cellular IL-22 source in PBMC exposed to *B. burgdorferi* 6.

As IL-1 induces IL-22 production in PBMC 6 and type I IFN has been demonstrated to inhibit IL-1β production 20,21, we assessed regulation of *B. burgdoferi*-induced IL-1β by IFN-α. In fact, coincubation with IFN-α significantly impaired release of IL-1β from PBMC exposed to *B. burgdorferi*. Likewise, IFN-α inhibited secretion of IL-8 (Fig. 2D), a prototypic IL-1-inducible chemokine 9. Data concur with the previous observation that IL-8 production by PBMC exposed to *B. burgdorferi* partly depends on IL-1 22.

## Discussion

Production of IFN-α in the context of infection by *B. burgdorferi* is, to a large part, dependent on TLR9 23 sensing unmethylated CpG motifs in microbial DNA. The present observation of IFN-α modulating production of IL-1β concurs with previous reports demonstrating, albeit in other experimental systems, reduced pro-IL-1β expression and/or maturation under the influence of type I IFN 20,21. Specifically, type I IFN increases murine susceptibility to *Candida albicans* by inhibiting IL-1β biological activity 20. Sufficient production of IL-1β in fact is critical for efficient host defence against a variety of microbial challenges 24. Notably, caspase-1-deficient mice display significantly higher bacterial loads in the initial phase of infection by *B. burgdorferi* 25.

STAT3-activating IL-22 mediates crucial anti-bacterial functions at biological barriers 11,26, including the skin 27. By modulating IL-22 expression, IFN-α may undermine bacterial clearance in an early-phase of infection. Moreover, on a further layer, by directing IL-22 signal transduction towards activation of STAT1, IFN-α has the capability to qualitatively modify tissue responses to IL-22 13,28. In this context, it is noteworthy that neutralization of STAT1-activating IFN-γ apparently reduces bacterial growth in experimental murine *B. burgdorferi* infection 29.

Data presented indicate the capability of IFN-α to curb induction of the anti-bacterial IL-1/IL-22 axis in response to the spirochaete. Along with, for example, anti-inflammatory properties of tick saliva, induction of type I IFN may be added to strategies ‘used’ by spirochaetes to subvert cytokine function thereby potentially affecting the course of early infection 30. Though observations presented confine to cultivated PBMC, this regulatory path may relate to the clinical course of *B. burgdorferi* infections as local IFN-α has been associated with the development of multiple *Erythema migrans* lesions 31, a phenomenon regarded as an indicator of early bacterial dissemination.
